# Synchronous volvulus of the transverse and sigmoid colon: a rare case of large bowel obstruction

**DOI:** 10.11604/pamj.2021.38.231.27470

**Published:** 2021-03-02

**Authors:** Abderrahim Samlali, Said Boussaidane, Asma Hamri, Youssef Narjis, Ridouan Benelkhaiat Benomar

**Affiliations:** 1 Department of Digestive Surgery, Hospital Ibn Tofaîl, University Hospital Center Mohammed VI, Marrakech, Morocco

**Keywords:** Volvulus, transverse, sigmoid, bowel obstruction, case report

## Abstract

Colonic volvulus usually occurs as a single event that can affect various parts of the colon. The usual sites affected being the sigmoid colon (75%) and the caecum (22%). The phenomenon of multiple sites simultaneously undergoing volvulus is an extremely rare occurrence. We report a rare case of simultaneous sigmoid and transverse colon volvulus in a 52-year-old female. The clinical presentation and the radiological findings were that of large bowel obstruction. A subtotal colectomy and colocolic anastomosis were performed and the postoperative period was uneventful. Though rare the development of transverse and sigmoid volvulus in the same patient must always be considered in the differential diagnosis, when dealing with recurrent intermittent abdominal pain or acute intestinal obstruction.

## Introduction

Colonic volvulus is the axial twisting of the colon on its vascular pedicle. The most common site is the sigmoid colon (75%) followed by the cecum (22%). Rare sites of colonic volvulus include the transverse colon (about 2%) and the splenic flexure (1-2%) [1]. Having a synchronous volvulus of multiple portions of the bowel is an exceedingly rare occurrence [2]. To our knowledge, few reports on simultaneous sigmoid and transverse colon have been published to date. Its rarity requires it to reported and brought to the attention to practicing surgeons as an unusual cause of large bowel obstruction. Prompt surgical intervention is key to decreasing the morbidity and mortality associated with this uncommon condition [2]. We report a case of a synchronous volvulus of the transverse and sigmoid colon that was treated such as a unique surgical emergency at the Department of Digestive Surgery, Hospital Ibn Tofaîl, University Hospital Center Mohammed VI of Marrakech.

## Patient and observation

**Figure 1:**
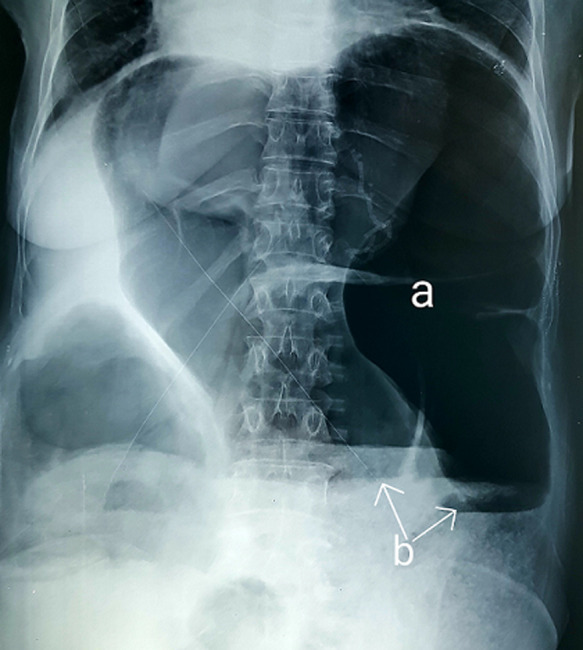
plain abdominal X-ray showing dilated colon (a) and air-fluid levels (b)

**Figure 2:**
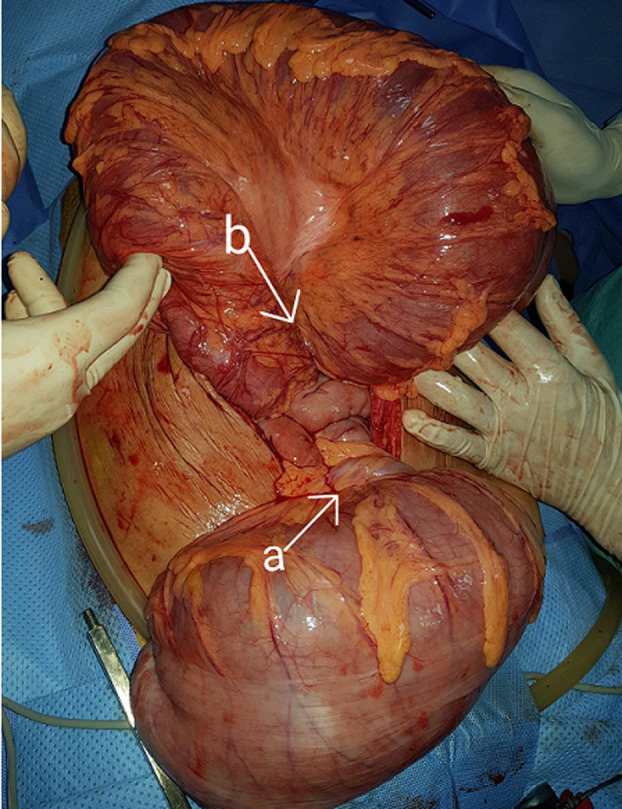
gross operative view of sigmoid volvulus (arrow a) and transverse colon volvulus (arrow b)

**Figure 3:**
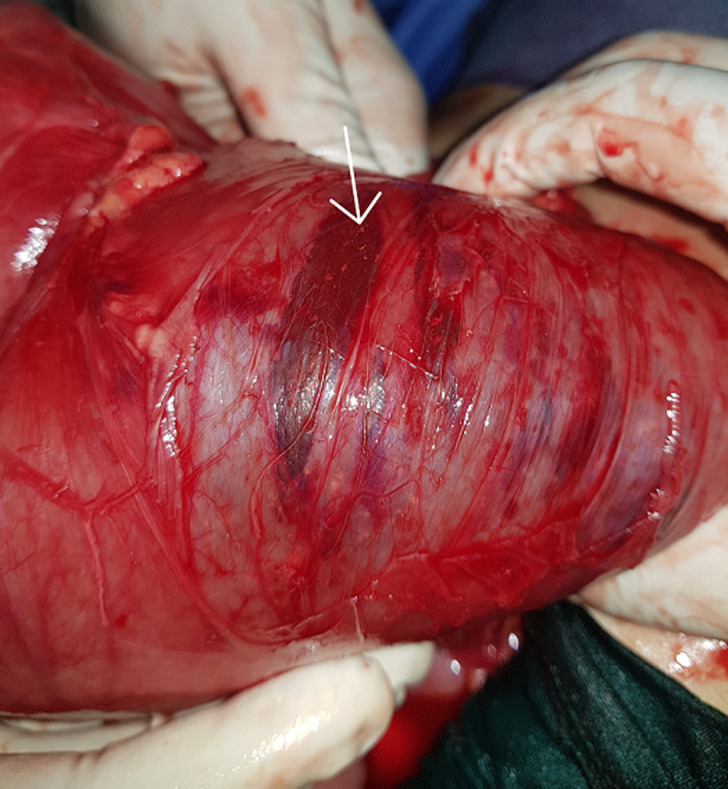
parietal thinning and pre-perforation area

A 52-year-old woman was admitted to surgical emergency with complete bowel obstruction of 3 days´ duration. She presented with no defecation and gas passing, abdominal pain, vomiting and grossly distended abdomen. The patient had no background history of chronic illnesses or previous operations. On examination, his vital signs were: temperature 37°C, pulse 95/minute, respiratory rate 25/minute, and blood pressure 105/60 mmHg. The patient´s abdomen was distended severely with generalized tenderness and diminished bowel sounds, rectal examination showed empty rectum without any intraluminal mass. Her emergent laboratory data were as follows: WBC 8600/mm^3^, and hemoglobin 13.5 g/dL, C-reactive protein (CRP) at 61 mg/l, and serum sodium and potassium levels were within normal limits. An abdominal X-ray revealed a grossly distended large bowel with air-fluid levels (Figure 1). A contrasted computed tomography (CT) scan revealed thick distended bowel loops with twisting of the mesenteric vessels associated with sigmoid volvulus. After a short period of resuscitation, an exploratory laparotomy was performed. Intraoperative findings revealed transverse colon volvulus and sigmoid volvulus associated with megacolon (Figure 2). Cecum, ascending, transverse colon and sigmoid were intensively distended and both transverse and sigmoid colon were twisted on their vascular pedicles with parietal thinning and some pre-perforation (Figure 3). Due to macroscopic evidence, intestinal resection and subtotal colectomy were performed followed by end-to-end colocolic anastomosis. The postoperative period was uneventful and the patient was discharged after 6 days. The histopathology results showed mucosal and submucosal congestion, chronic inflammation, compatible with volvulus and no malignancy or dysplasia was found.

## Discussion

Volvulus is described as abnormal twisting of bowel along its mesenteric axis leading to closed-loop obstruction. It stops venous return and compromises arterial supply leading to ischemia [3]. Volvulus itself is an unusual cause of intestinal obstruction accounting for ~5% of cases of gastrointestinal obstruction and 10-15% of large bowel obstruction. The most common locations for colonic volvulus are the sigmoid colon (75%), caecum (15%), transverse colon (3%) and splenic flexure (2%) [2]. The phenomenon of multiple sites simultaneously undergoing volvulus is a very rare event [2]. The occurrence of concurrent sigmoid and transverse colon volvulus is extremely rare. There is paucity of information in the literature regarding synchronous sigmoid and transverse colon volvulus [4]. There are some causes for the fact that transverse colon is a rare location for colonic volvulus. Besides beneficial anatomical position of transverse colon, short mesocolon and colonic flexure keep this part of colon in its anatomical location. Some mechanical and physiological factors like megacolon, constipation, distal colon obstruction, adhesion band and previous surgery have been considered as etiologies of colonic volvulus. However, redundancy and non-fixation are the two essential factors in forming colonic volvulus [3-5]. Our patient had redundant colon with narrow sigmoid and transverse mesoscopic parietal attachments.

The diagnosis of this condition is usually made at laparotomy despite a thorough history, examination and appropriate radio logical investigations [2]. There are two clinical presentations of colonic volvulus that have been described: acute fulminant and subacute progressive. Acute volvulus is characterized by nausea, vomiting, marked leukocytosis, acute abdominal pain, signs of peritoneal irritation and sometimes bloating. The subacute presentation is accompanied by less nausea and vomiting, mild abdominal pain, abdominal distention without significant peritonitis, and normal or slightly elevated leukocyte counts [3-6]. Lab study usually shows mild or no leukocytosis and no fever in the early stages of the disease. Abdominal x-ray and CT-scan can be helpful to confirm the diagnosis like our case [5-7]. In the absence of clinical and radiological signs of necrosis or perforation, the initial management of volvulus involves colonoscopic derotation and decompression followed by semi-elective resection and anastomosis after optimizing the patient [4-8]. However, in the cases of colonoscopy failure, necrosis or perforation signs surgery is required with ostomy or anastomosis depending on the bowel status and patient stability. A detorsion without resection of the colon is associated with high recurrence rates [4]. Although transverse and sigmoid colon volvulus is a rare case of bowel obstruction, it is advised to consider it in the differential diagnosis of abdominal pain and recurrent bowel obstruction to prevent unfortunate outcomes.

## Conclusion

Synchronous volvulus of the sigmoid and transverse colon is an extremely rare clinical entity. Its diagnosis can be difficult and management effectiveness remains controversial. Prompt recognition with emergency intervention constitutes the key to a successful outcome.
